# Impact of the COVID-19 pandemic on COPD exacerbations in Japanese patients: a retrospective study

**DOI:** 10.1038/s41598-024-53389-2

**Published:** 2024-02-02

**Authors:** Toshihiko Nishioki, Tadashi Sato, Akifumi Okajima, Hiroaki Motomura, Tomohito Takeshige, Junko Watanabe, Toshifumi Yae, Ryo Koyama, Kenji Kido, Kazuhisa Takahashi

**Affiliations:** 1 Department of Respiratory Medicine, Juntendo University Nerima Hospital, 3-1-10, Takanodai, Nerima-ku, Tokyo, 177-8521 Japan; 2 Department of Respiratory Medicine, Juntendo University Graduate School of Medicine, 3-1-3, Hongo, Bunkyo-ku, Tokyo, 113-8431 Japan

**Keywords:** Diseases, Medical research

## Abstract

Various infection control measures implemented during the coronavirus disease (COVID-19) pandemic have reduced the number of respiratory infections, which are the most common cause of chronic obstructive pulmonary disease (COPD) exacerbations. Here, we investigated whether infectious disease prevention during the COVID-19 pandemic reduced COPD exacerbations and the characteristics of patients exhibiting exacerbations before and during the COVID-19 pandemic. We included outpatients and inpatients with moderate or severe COPD exacerbations who required systemic steroids between April 1, 2018 and March 31, 2022. Their medical records were retrospectively compared and analyzed in 2-year intervals (before and during the COVID-19 pandemic). During the 4-year observation period, 70,847 outpatients and 2,772 inpatients were enrolled; 55 COPD exacerbations were recorded. The number of COPD exacerbations decreased from 36 before to 19 during the COVID-19 pandemic. Regarding the characteristics of patients with exacerbations, the % forced expiratory volume in one second (52.3% vs. 38.6%, *P* = 0.0224) and body mass index (BMI) (22.5 vs. 19.3, *P* = 0.0127) were significantly lower during the COVID-19 pandemic than before the pandemic. The number of COPD exacerbations during the pandemic decreased. Additionally, the tendency for a reduction in COPD exacerbation was greatest in patients with preserved lung function or above-standard BMI patients.

## Introduction

With the emergence of coronavirus disease (COVID-19), various infection prevention measures have been widely implemented. Specifically, avoiding leaving the house, wearing masks, using hand sanitizers, and avoiding the three Cs (closed spaces, crowded spaces, and closed-contact settings) have been widely adopted. As a result, the incidence of both COVID-19 and other respiratory infections has reportedly decreased^1,2^. Other studies have also reported that wearing masks against COVID-19 may have reduced the number of influenza, enterovirus, and all-cause pneumonia cases^3^.

Chronic obstructive pulmonary disease (COPD) exacerbations are significant conditions that worsen the quality of life and respiratory function of patients with COPD and worsen their prognosis^4^. Although several factors can cause exacerbation of COPD, respiratory infections are the most common, accounting for 78% of all cases^5^. Viral infections, including rhinoviruses, influenza viruses, and respiratory syncytial viruses*,* are the most common causes of these infections^6,7^. However, in actual medical practice, we experienced a marked decrease in hospital visits due to acute upper respiratory infections during the COVID-19 pandemic. We assumed that COPD exacerbations might have decreased due to a decrease in respiratory infections. Therefore, we believe it would beneficial to assess whether common infection prevention measures are effective in preventing COPD exacerbations, and which patients are safe and which are still more prone to exacerbations and require more careful attention. Although some reports have reported this decreasing trend, most were based on short-term, inpatient-only observations and comparisons based on database analyses using only disease registries^8^. No studies have reported the characteristics of patients with COPD for whom infection control measures are effective. As COPD exacerbation can be seasonal, conducting studies with a uniform observation period would be desirable. Furthermore, with the recent spread of inhalation medications, COPD exacerbation does not necessarily require hospitalization; in many cases, it can be effectively treated on an outpatient basis. Therefore, we aimed to compare whether measures taken against COVID-19 reduced COPD exacerbations all year round for inpatients and outpatients, while confirming results of each case. Additionally, we aimed to find previously unreported characteristics of patients whose COPD exacerbation was influenced by COVID-19 pandemic prevention measures.

## Results

### COPD exacerbations decreased during the COVID-19 pandemic

During the 4 years, 70,847 outpatients and 2,772 inpatients were included in the study. In the fiscal years 2018, 2019, 2020, and 2021, there were 16,992, 17,285, 17,283, and 19,287 outpatients, respectively, and 670, 710, 659, and 733 inpatients, respectively. Among them, 55 COPD exacerbations (12 multiple exacerbations in 5 patients) met the inclusion criteria. In patients with exacerbations, systemic steroid durations and doses ranged from 3 to 15 days and 10–400 mg/day of prednisone equivalent. In the 2018–2019 fiscal year (April 1, 2018, to March 31, 2020), before the COVID-19 pandemic, there were 36 cases, while in the 2020–2021 fiscal year (April 1, 2020, to March 31, 2022), during the pandemic, there was a marked decrease to 19 cases. The number of outpatients was 14 vs. 6 and that of inpatients was 22 vs. 13, both of which were reduced by approximately half.

### Lower %FEV_1_ and BMI were found in patients who had exacerbations during the COVID-19 pandemic

There was a decrease in the number of inpatients and outpatients all year round due to the COVID-19 pandemic. The characteristics of the patients who had exacerbations were compared before and during the COVID-19 pandemic to evaluate the characteristics of the patient population with reduced exacerbations.

There were no significant differences in age: 75.1 vs. 73.8 years (*P* = 0.6791), sex (male/female): 27/9 vs. 17/2 (*P* = 0.2952), and number of people living together: 2.1 vs. 2.1 (*P* = 0.9734). Regarding characteristics that could directly affect the frequency of exacerbations, no significant differences were found in the number of concomitant inhaled medications [long-acting muscarinic antagonist (LAMA), long-acting β-agonists (LABA), and inhaled corticosteroids (ICS)]:1.3 vs. 1.9 (*P* = 0.0589), smoking history (pack-years): 64 vs. 66 (*P* = 0.8248), the proportion who continued smoking at exacerbations: 33.3% vs 36.8% (*P* = 0.7947), and mortality rate: 11.1% vs 15.8% (*P* = 0.6823). In contrast, % forced expiratory volume in one second (%FEV_1_): 52.3% vs. 38.7% (*P* = 0.0224) and body mass index (BMI): 22.5 vs. 19.4 (*P* = 0.0127) were significantly lower during the COVID-19 pandemic (Table 1).

**Table 1:** Comparison of characteristics of patients with COPD exacerbation before and during the COVID-19 pandemic.

	2018–2019 fiscal year	2020–2021 fiscal year	p-value
	Before COVID-19 (n = 36)	During COVID-19 (n = 19)	
General information			
Age	75.1 (50–95)	73.8 (60–90)	0.6791
Men/female	27/9	17/2	0.2952
BMI (kg/m^2^)	22.5 (14.4–32.8)	19.4 (13.1–29.7)	**0.0127**
Pack-year	64 (22–150)	66 (23–128)	0.8248
Current smoke	12 (33.3%)	7 (36.8%)	0.7947
Number of people living together	2.1 (1–4)	2.1 (1–4)	0.9734
Death (%)	4 (11.1%)	3 (15.8%)	0.6823
Examinations			
Leukocyte (/μL)	10,341 (4100–29,000)	10,974 (4500–21,700)	0.3590
Eosinophil (/μL)	120 (0–680)	216 (0–1146)	0.1323
CRP (mg/dL)	6.86 (0.04–31.03)	3.01 (0.00–15.50)	0.0916
FEV_1_/FVC (%)	45.4 (23.6–68.4)	38.4 (28.6–57.5)	0.0614
%FEV_1_ (%)	52.3 (22.9–106)	38.7 (19.1–89.1)	**0.0224**
Therapy			
LAMA	17 (47.2%)	13 (68.4%)	0.1333
LABA	18 (50.0%)	13 (68.3%)	0.1902
ICS	11 (30.6%)	10 (52.6%)	0.1091
Total number of inhaled drugs	1.3 (0–3)	1.9 (0–3)	0.0589
Home oxygen therapy	10 (27.8%)	4 (21.0%)	0.7486

*BMI* body mass index, *CRP* C-related protein, *FEV1* forced expiratory volume in one second, *FVC* forced vital capacity, *LAMA* long-acting muscarinic antagonist, *LABA* long-acting β-agonists, *ICS* inhaled corticosteroids.

Significant values are given in bold.

**Figure 1:**
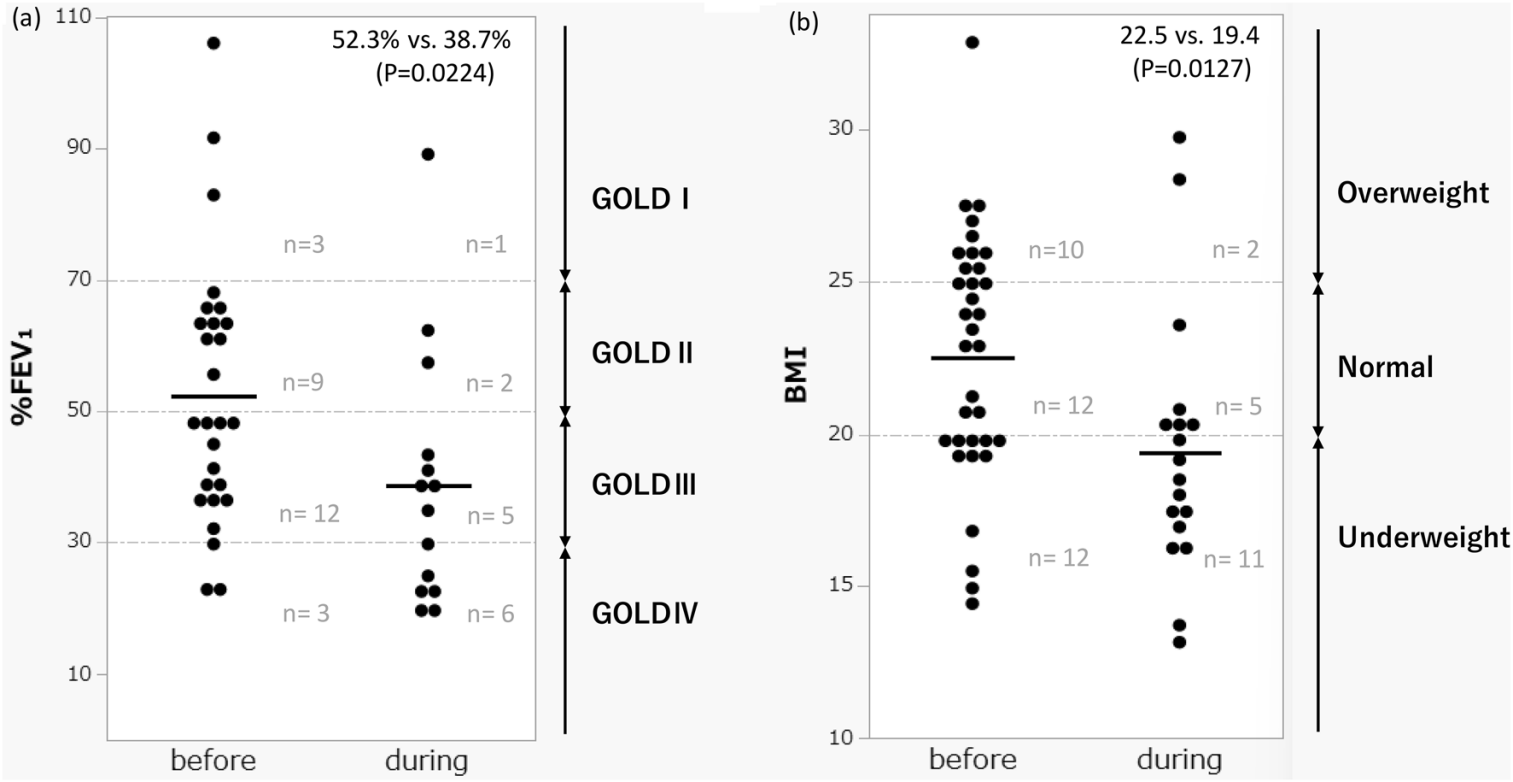
Distribution of %FEV_1_ and BMI in patients with COPD exacerbations before and during COVID-19 pandemic. Significant differences in %FEV_1_ and BMI were found between patient groups before and during the COVID-19 pandemic. Both mean values decreased during the pandemic. (**a**) In the distribution of %FEV_1_, in GOLD I, II, and III centered around %FEV_1_ 60%, exacerbations decreased to less than half before and during the pandemic, exceeding the decreasing trend in the overall population. In contrast, there was an increase in GOLD IV scores. (**b**) Similarly, for BMI, exacerbations decreased by more than half for BMI ≥ 20, mainly around 25. Moreover, there was no reduction in exacerbations during the pandemic at BMI below 20. *FEV*_*1*_ forced expiratory volume in one second, *GOLD* global initiative for chronic obstructive lung disease, *BMI* body mass index.

In the patient distribution chart, the number of exacerbations tended to decrease predominantly during the COVID-19 pandemic in the group with relatively preserved %FEV_1_ and BMI (%FEV_1_ approximately 60%, BMI approximately 25 kg/m^2^). According to the Global Initiative for Chronic Obstructive Lung Disease (GOLD) classification, the number of patients with COPD exacerbations decreased by 66% (3 vs. 1) in GOLD I, 77% (9 vs. 2) in GOLD II, and 58% (12 vs. 5) in GOLD III during COVID-19, whereas GOLD IV showed a 100% increase (3 vs. 6). When BMI was classified as underweight (< 20) and overweight (25 ≤), the number of exacerbations decreased markedly in those with a BMI ≥ 20 (22 vs. 7), whereas no significant decrease was observed in those with a BMI of < 20 (12 vs. 11) (Fig. 1).

## Discussion

There was a decrease in COPD exacerbations in the 2020–2021 fiscal year compared to the 2018–2019 fiscal year. The difference between the two periods was the COVID-19 pandemic, and the most influential cause was the infection prevention measures including wearing masks. Medical factors other than infection control that may have reduced COPD exacerbations include improved smoking cessation rates associated with increased health motivation, treatment for COPD, and adherence to medications^9^. We found no significant difference in the rate of smoking cessation. Moreover, the total number of inhaled drugs, such as LAMA/LABA/ICS, which could suggest an intensification of treatment, remained almost the same. There was no apparent change in medication adherence in the medical records, at least for patients who had been continuously monitored in our department before the exacerbation. Additionally, there were reports that air pollutant concentrations have decreased during the lockdown period, associated with economic stagnation, and that the number of hospitalizations for COPD exacerbations and Asthma attacks has also decreased^10,11^. However, other researchers have reported that medical factors other than infection control have little impact^12^. Thus, because the main cause of COPD exacerbation is infection, we assumed these environmental effects including social factors remain controversial. Another factor was the reluctance to visit a doctor. There are reports of a temporary decrease in hospital visits due to the belief that hospital visits pose an infection risk following the COVID-19 pandemic^13^. However, in our hospital, the emergency department is mainly responsible for COVID-19 patients, and the total number of inpatients and outpatients in the respiratory department did not decrease at least, rather had a slight increase due to the expansion of the size of the practice. Additionally, patients who required systemic steroid treatment, such as those with moderate or severe symptoms, were assumed to be less likely to refrain from visiting the doctor. Hence, in this study, we considered that refraining from hospital access would have little effect on the number of COPD exacerbations. Therefore, it can be inferred that the measures taken against COVID-19 reduced the number of respiratory tract infections, which in turn reduced the incidence of COPD exacerbations.

A comparison of the patient population with COPD exacerbations before and during COVID-19 showed that patients who had exacerbations during COVID-19 had significantly lower %FEV_1_ and BMI. It is known that the milder the progression of COPD airway obstruction, the less frequent the exacerbations; the higher the BMI, the less likely COPD exacerbations occur^14–16^. Infection is the most common cause of the development of COPD exacerbations; however, it is not only influenced by environmental factors, including infection, but also by patient factors, such as frailty, metabolic syndrome, immune, and genetic factors^17^. Although no precise mechanism has been elucidated, %FEV_1_ and exacerbations are interrelated. The more exacerbations that occur, the more airway narrowing associated with chronic inflammation progresses. The more lung function declines, more likely exacerbations will occur due to decreased infection defense mechanisms in the patient^4,18^. The same was true for BMI. Although it is difficult to determine the full distribution of fat/lean body mass throughout the body, more exacerbations occur, and more muscle mass is lost with systemic inflammation. Additionally, more respiratory muscles are reduced, which can lead to more frequent exacerbations^19,20^.

In addition, the BODE index; B: BMI ≥ 21; O: Obstruction %FEV_1_ ≥ 65%; D: Dyspnea modified medical research council dyspnea scale ≤ 1; E: Exercise capacity ≥ 350 m on a 6-min walk, which is widely known as a mortality risk assessment for COPD, can be used as a risk assessment for exacerbations^21,22^. The significant predictors of COPD exacerbations are low BMI, low pulmonary function, poor health-related quality of life, and a history of previous exacerbations. Moreover, %FEV_1_ and BMI are clinical parameters that can be obtained in daily practice, are elements with objective criteria, and play a significant role as predictors of COPD exacerbation.

Compared to patients with COPD in Western countries, Japanese patients are older, have a lower BMI (19–24 vs. 26–28), are more emphysema-dominant (90%), and have fewer comorbidities, such as cardiovascular disease and metabolic syndrome^23,24^. Nevertheless, Japanese patients have a lower frequency of exacerbations. Moreover, the exacerbation rate of COPD in Japan is low compared to other Asian countries, therefore, rather than racial disparities, universal health insurance system is thought to be the cause^25^. However, this study, conducted in Japan, also found that few patients had multiple exacerbations. Therefore, daily disease control may also be important in the prevention of COPD exacerbations. Additionally, regarding the possibility of random errors occurring due to sample size, the BMI data was normally distributed, and the 95% confidence interval for the population mean calculated from pre-epidemic data was 21.09–23.91, unchanged from previous reports. This was similar to previous reports that exacerbation is more likely to occur with low BMI and low respiratory function which showed a significant difference even with this sample size. Although the possibility of insufficient detection of minute differences cannot be denied, it is assumed that these our result are valid.

In combination with the results of this study, it is possible that infection control measures may have been more effective in preventing exacerbations in patients with relatively infrequent COPD exacerbations, such as those with a maintained %FEV_1_ or above-standard BMI. We consider that patients with advanced COPD or those who are emaciated and relatively prone to COPD exacerbations might be unable to prevent respiratory infections and exacerbations even with infection control measures, resulting in a relative decline in the mean values of %FEV_1_ and BMI during the COVID-19 pandemic. These patients may also require further COPD exacerbation prevention that includes more thorough infection preventions and measures for unexplained exacerbations accounting for about one-third which does not rely solely on infection control.

**Figure 2:**
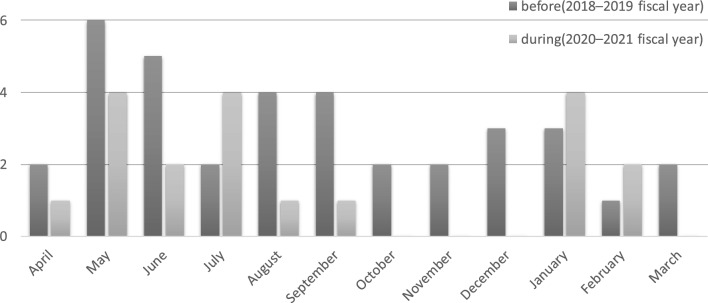
Number and annual distribution of patients with COPD exacerbations before and during the COVID-19 pandemic. Distribution of the number of exacerbations per month throughout the year. No significant changes are observed in the seasonal distribution of COPD exacerbations before the COVID-19 pandemic; 2018–2019 fiscal year, and during the COVID-19 pandemic; 2020–2021 fiscal year.

In this study, each 2-year survey period was compared with uniform seasons to evaluate the results throughout the year and to eliminate the effects of the seasonal disparities. We found no significant difference in seasonal variation before and during COVID-19 (Fig. 2). During these 4 years, there were 1st-6th waves of pandemics, and the incidence in our municipality ranged from 1 to 1508 cases/100,000 persons/month, but there was also no clear correlation with the number of patients with COPD exacerbations.

Nonetheless, a limitation of this study is that it was conducted on patients treated by respiratory medicine specialists at a university hospital and did not include those treated by primary care physicians. However, the fact that most patients underwent spirometry and computed tomography at the time of COPD diagnosis is a strength of this study. Further studies should be conducted in prospective multicenter settings. In addition, because we did not exclude patients with asthma complications, it is possible that we were unable to completely exclude asthma attacks. However, the concept of Asthma and COPD Overlap has become widespread, and even after establishing a diagnosis of COPD in clinical practice, it is difficult to clearly distinguish whether asthma complications are present in many cases. From this point of view, we believe that we were able to conduct a study in line with actual clinical practice while making a more reliable diagnosis of the presence or absence of COPD.

In conclusion, during the COVID-19 pandemic, the number of COPD exacerbations decreased in both inpatient and outpatient settings. The reduction was more substantial in patients with relatively preserved %FEV_1_ (GOLD I–III) or in less emaciated patients (BMI ≥ 20). It is suggested that infection control measures may have been effective in preventing exacerbations in COPD patients with relatively preserved BMI and lung function.

## Methods

### Study design

Outpatients and inpatients diagnosed with moderate or severe COPD exacerbation and treated with systemic steroids between April 1, 2018 and March 31, 2022, at the Department of Respiratory Medicine, Juntendo University Nerima Hospital, were included. The ethics committee of the Juntendo University Nerima Hospital approved the study protocol (number E22-0246-N01). All experiments were performed in accordance with the relevant guidelines and regulations. Requirement for informed consent was waived by ethics committee of the Juntendo University Nerima Hospital due to the retrospective nature of the study.

Eligible COPD patients had to be diagnosed with COPD by their physicians based on the descriptions in the GOLD statement, have a smoking history of > 20 pack-years, have a respiratory function test under normal conditions with a forced expiratory volume in one second/forced vital capacity (FEV_1_/FVC) < 70% (excluding cases with a high risk of pneumothorax or poor oxygenation), and have findings consistent with COPD on computerized tomography chest imaging. For consistency, the diagnosis of exacerbation and whether steroids should be administered and duration and dose were based on the attending physician’s judgement and were confirmed from the medical records.

Among outpatients with COPD, we selected those who were prescribed steroids for COPD exacerbations and excluded those who were prescribed steroids for other diseases (e.g., for antiemetic purposes for lung cancer chemotherapy or to prevent anaphylaxis). For inpatients, we selected those who were either registered as (i) having COPD, or overlap between asthma and COPD, or exacerbation of COPD during hospitalization, or (ii) had COPD included in the hospital discharge summary, who met the above COPD conditions and were treated with steroids for COPD exacerbations based on their discharge summary and medical records. Additionally, we included patients matching the above criteria, even those with chest ground-glass opacities or consolidation and asthma complications to align the study with actual clinical practice. Exclusion criteria were patients who did not meet the above requirements for COPD.

Data were collected retrospectively for a total of 4 years to eliminate disparities between seasons and years. Basic patient information, respiratory function test results obtained outside of periods of exacerbation, long-term control medications, blood test results at exacerbation, and treatment outcome were recorded. The first emergency declaration for the COVID-19 pandemic in Japan was issued on April 7, 2020, therefore, people’s infection-prevention lifestyle was assumed to have become widely established since then. The last priority preventative measures ended on March 21, 2022; thus, it is assumed that people gradually returned to their normal lives after that. These 2 years; 2020–2021 fiscal year (April 1, 2020, to March 31, 2022) were set as during the COVID-19 pandemic, the immediately preceding 2 years; 2018–2019 fiscal year (April 1, 2018, to March 31, 2020) were set as before the COVID-19 pandemic, and data collected on a medical record basis were compared retrospectively.

### Statistical analysis

Continuous variables are described as means and ranges from minimum to maximum, while categorical variables are expressed as numbers and percentages. Comparison between groups of the quantitative variables of age, BMI, pack-year, number of people living together, blood leukocyte count, blood eosinophil count, C-reactive protein, FEV_1_/FVC, and %FEV_1_was performed using the Student's t-test and by the Wilcoxon’s rank-sum test for independent samples. The chi-square test or Fisher’s exact test was used to examine categorical data, including sex, current smoking, death, use of LAMA, LABA, and ICS, and home oxygen therapy. A two-tailed *P* ≤ 0.05 was considered statistically significant. All statistical analyses were performed using the JMP Pro software version 16 (SAS Institute Inc., Cary, North Carolina, USA).

## Data Availability

The datasets used and/or analyzed during the current study available from the corresponding author on reasonable request.
